# ROS-Generating Hyaluronic Acid-Modified Zirconium Dioxide-Acetylacetonate Nanoparticles as a Theranostic Platform for the Treatment of Osteosarcoma

**DOI:** 10.3390/nano13010054

**Published:** 2022-12-22

**Authors:** Giovanna Chianese, Ines Fasolino, Chiara Tramontano, Luca De Stefano, Claudio Imparato, Antonio Aronne, Luigi Ambrosio, Maria Grazia Raucci, Ilaria Rea

**Affiliations:** 1 Unit of Naples, National Research Council, Institute of Applied Sciences and Intelligent Systems, 80131 Naples, Italy; 2 National Research Council, Institute of Polymers, Composites and Biomaterials, 80131 Naples, Italy; 3 Department of Pharmacy, University of Naples Federico II, 80131 Naples, Italy; 4 Department of Chemical, Materials and Production Engineering, University of Naples Federico II, 80125 Naples, Italy

**Keywords:** hybrid zirconia, nanoparticles, functionalization, photoluminescence, reactive oxygen species, cancer therapy, osteosarcoma

## Abstract

Materials that are able to produce free radicals have gained increasing attention for environmental and biomedical purposes. Free radicals, such as the superoxide anion (O_2_^•−^), act as secondary messengers in many physiological pathways, such as cell survival. Therefore, the production of free radicals over physiological levels has been exploited in the treatment of different types of cancer, including osteosarcoma (OS). In most cases, the production of reactive oxygen species (ROS) by materials is light-induced and requires the use of chemical photosensitisers, making it difficult and expensive. Here, for the first time, we propose photoluminescent hybrid ZrO_2_-acetylacetonate nanoparticles (ZrO_2_-acac NPs) that are capable of generating O_2_^•−^ without light activation as an adjuvant for the treatment of OS. To increase the uptake and ROS generation in cancer cells, we modify the surface of ZrO_2_-acac NPs with hyaluronic acid (HA), which recognizes and binds to the surface antigen CD44 overexpressed on OS cells. Since these nanoparticles emit in the visible range, their uptake into cancer cells can be followed by a label-free approach. Overall, we show that the generation of O_2_^•−^ is toxic to OS cells and can be used as an adjuvant treatment to increase the efficacy of conventional drugs.

## 1. Introduction

The functional properties of metal-oxide semiconductors, such as photocatalytic activity [1], photoluminescence [2], and chemical and thermal stability [3], are prospective tools for biomedical applications [4,5]. Among the most common metal-oxide semiconductors used in biomedicine, zirconium oxide, ZrO_2_, has gained increasing attention in theranostics. ZrO_2_ has the optical property of semiconductors, with strong absorption in the ultraviolet region and emission in the visible range. Most recently, it has been employed in catalysis [6], dentistry [7], biosensing [8,9], and photodynamic therapy [10]. The main intrinsic defects of ZrO_2_, the oxygen vacancies, play a key role in the generation and stabilization of free radicals on the material surface. This property has made ZrO_2_ a well-known catalyst for environmental purposes due to its ability to convert CO_2_ or to act as a radical initiator in the oxidative degradation of phenanthrene [11,12,13]. However, the ability to generate the superoxide radical anion (O_2_^•−^) is also useful for the treatment of cancer, where the excess of O_2_^•−^ can damage cells and induce apoptosis. The anion O_2_^•−^ is a reactive oxygen species (ROS) naturally produced by the cellular oxidative metabolism, and it is involved as a secondary messenger in various physiological pathways, such as cell survival [14]. ROS are constantly produced by the organism and scavenged by the natural antioxidant mechanism, which binds to free radicals, inhibiting their action. The generation of free radicals has been exploited in a series of anticancer strategies [15,16] since the accumulation of free radicals over the physiological level activates regulated cell death (RCD) programs, such as apoptosis, necroptosis, and ferroptosis [17]. Therefore, in recent years, metal-oxide semiconductors with the capability of producing ROS in cancer cells have gained increasing attention. In particular, when ZrO_2_ is produced by a sol-gel route in the presence of acetylacetone (acac), a hybrid material capable of producing and stabilizing free radicals without light irradiation can be obtained [18]. The presence of acac in the hybrid ZrO_2_ lowers the energy for the generation of oxygen vacancies, promoting the adsorption of molecular oxygen and its reduction to O_2_^•−^ [19,20]. This material shows intriguing photoluminescent properties in the visible range [21], which, combined with the capability of producing ROS, make the hybrid ZrO_2_ a powerful theranostic material for cancer therapy. In particular, the generation of ROS has been used as a synergistic process in the treatment of osteosarcoma (OS) to increase the efficacy of chemotherapeutic drugs [22]. OS is a bone cancer occurring most often in children and young adults between the age of 10 and 20 years [23]. The chemotherapeutic agents employed in the treatment of OS include cisplatin, doxorubicin, and methotrexate, which have been reported to kill cancer cells by inducing ROS-dependent apoptosis [24]. However, the neutralization of ROS by the antioxidant mechanism reduces the efficacy of conventional therapeutic approaches [25]. Therefore, increasing the levels of ROS produced by conventional drugs is a promising strategy for enhancing their efficacy and reducing the dosage regimen. In this scenario, the use of hybrid ZrO_2_-acac nanoparticles (ZrO_2_-acac NPs) spontaneously producing ROS provides unique features for the treatment of OS. They possess the peculiar characteristic of generating ROS without light irradiation and at room temperature, due to the combined effects played by the ligand-to-metal charge transfer process and the defectivity of the material [26]. It was demonstrated that, in such hybrid materials, the production of stable ROS requires the presence of superficial oxygen vacancies which leave electrons and reduced metallic centers coordinatively unsaturated on the surface [17,21]. Moreover, the photoluminescence properties of ZrO_2_-acac may allow for the monitoring of the internalization of the NPs in OS cells with a label-free approach. Overall, the great advantage of using ZrO_2_-acac NPs as an adjuvant in OS treatment is the possibility of modifying their surface to recognize cancer cells specifically, increasing the uptake and ROS generation in malignant cells rather than healthy ones. Numerous studies have reported the overexpression of surface molecules on the OS cells, including CD44, the major hyaluronic acid (HA) receptor [27]. The binding between CD44 and HA has been proven to be involved in multidrug resistance [28], tumor progression, metastasis, and proliferation [22]. Numerous ROS-generating NPs targeting CD44^+^ cells have been proposed [23,29,30], but, to the best of our knowledge, none of them combines imaging and therapy in a label-free and light-independent approach, such as our ZrO_2_-acac NPs.

Here, we propose a theranostic platform for the treatment of OS made of photoluminescent hybrid ZrO_2_-acac NPs generating ROS at room temperature and without light activation. We show that the O_2_^•−^ generated by ZrO_2_-acac NPs, obtained by the ultrasonication of the corresponding sol-gel-derived micrometric powders, has a toxic effect on SAOS-2 cells. To promote the accumulation of ROS in these cells, we modify the surface of ZrO_2_-acac NPs with HA (ZrO_2_-acac NPs-HA), which is recognized and bound to CD44 on SAOS-2 cells, enhancing the NP uptake in the targeted cells. The unique photoluminescence properties of this material in the visible range are preserved in the NPs, allowing us to trace their internalization in cells without the need for external fluorophores. We demonstrate that the production of ROS by hybrid ZrO_2_-acac NPs-HA kills SAOS-2 cells and can be considered as an adjuvant treatment for increasing the efficacy of conventional chemotherapeutic drugs.

## 2. Materials and Methods

### 2.1. Materials

Zirconium(IV) propoxide (70 wt% in 1-propanol), acetylacetone (Hacac), 1-propanol, absolute ethanol, 3-aminopropyltriethoxysilane (APTES), N-(3-Dimethylaminopropyl)-N′-ethyl carbodiimide (EDC), Phalloidin–Atto 594, N-hydroxysuccinimide (NHS), heat-inactivated fetal bovine serum (FBS), streptomycin, and penicillin were purchased from Merck, DE. Sodium hyaluronate (55 KDa) (HA) was provided by Altergon SRL (Avellino, Italy). 2,7-Dichlorofluorescin diacetate (DCFH-DA) was purchased from Cabru, IT. All the reagents were used without further purification. Hank Balanced Salt Solutions (HBSS) were purchased from Gibco (Milan, IT). Alamar Blue assay was purchased from Serotec, (Milan, IT).

### 2.2. Production of ROS-Generating ZrO_2_-acac NPs

ZrO_2_-acetylacetonate was synthesized by a sol-gel route according to previous work as a gel coarse powder [12,17]. Briefly, the hybrid gel was obtained by adding acac and 1-propanol to Zr(IV) propoxide in a ratio of 0.5 mol acac per mol of Zr, followed by the addition of a distilled water–propanol solution and letting the gel dry in a ventilated oven at 40 °C. Twenty milligrams of the powder were dispersed in 40 mL of ethanol and subjected to ultrasounds by an ultrasonic homogenizer (Bandelin, HD3200) equipped with an MS73 probe. Ultrasounds were applied in pulse mode (30 s pulse and 30 s pause) for 2 h to reduce the particle size, guaranteeing that the high temperature does not disrupt the matrix [31]. The material was then diluted to 200 mL and left to settle overnight. The supernatant of the sedimentation was centrifuged at 15,000 rpm and 15 °C for 10 min, and the NPs were recovered by pulling the pellets in a unique sample. The NPs (ZrO_2_-acac NPs) were weighed and stored in ethanol at a concentration of 1 mg mL^−1^ until further use.

### 2.3. Functionalization of ZrO_2_-acac NPs

ZrO_2_-acac NPs were settled in ethanol and amino-functionalized in a 10% APTES solution for 2 h under mild stirring. After the reaction, the NPs were centrifuged at 15,000 rpm and 15 °C for 10 min, and the supernatant with unreacted APTES was discarded. Ethanol was used for replacing the supernatant three times to remove all the unreacted APTES, and then the NPs (ZrO_2_-acac NPs-APT) were washed twice in water for the subsequent reaction. A solution containing 5 mg of hyaluronic acid, 38 mg of EDC, and 27 mg of NHS was prepared and used to suspend ZrO_2_-acac NPs-APT. The chemical reaction proceeds for 90 min under magnetic stirring and room temperature. In the end, the magnetic bar was removed, and the NPs dispersion was centrifuged at 15,000 rpm and 15 °C for 10 min to ward off residues. The NPs were resuspended several times in distilled water and centrifuged to remove any unreacted residue, and they were stored at room temperature in distilled water until future use.

### 2.4. Characterization of Bare and Modified ZrO_2_-acac NPs

The morphology of ZrO_2_-acac NPs was investigated using an FEI Tecnai 12 transmission electron microscope (FEI Company; Hillsboro, OR, USA). The NPs (0.1 mg mL^−1^ in water) were deposited on a standard copper grid (100 mesh) covered with a Formvar film. After 10 min, the drop was removed, and the grid was air-dried overnight at room temperature. The mean size of the ZrO_2_-acac NPs was estimated from the distribution of the Gaussian fit obtained by ImageJ software (rel. 1.53k, NIH, Bethesda, MD, USA).

To perform the photoluminescence (PL) analysis of the ZrO_2_-acac NPs, 20 μL of NPs dispersed in distilled water (1 mg mL^−1^) was deposited on a silicon wafer and left to dry at room temperature. The light emission of the samples was excited using a continuous wave He-Cd laser at 325 nm (KIMMON Laser System). PL spectra were collected at a normal incidence to the surface of samples through a fiber, dispersed in a spectrometer (Princeton Instruments, SpectraPro 300i), and detected using a Peltier cooled charge-coupled device (CCD) camera (PIXIS 100F). The laser line was cut by placing a long pass filter with a nominal cut-on wavelength of 350 nm at the monochromator inlet.

Fluorescence images of ZrO_2_-acac NPs were acquired using LAS X (Leica Application Suite; rel. 3.0.13) software linked to the Leica Camera DFC7000T of a Leica AF6000LX microscope (Leica Microsystems, Mannheim, Germany). Fluorescence images were obtained using an excitation wavelength of 365 nm and 470 nm.

The absorption spectra of the ZrO_2_-acac NPs and ZrO_2_-acac NPs-HA were acquired at concentrations of 0.125, 0.25, and 0.5 mg mL^−1^ in distilled water using a Jasco V-730 UV-Vis double-beam spectrophotometer (Jasco Inc., Easton, PA, USA). The investigation range was set to 250–600 nm, and measurements of water as the blank were subtracted from the samples.

The fluorescence emission spectra of the NPs were acquired by a JASCO FP-8200 (JASCO, Tokyo, Japan) spectrofluorometer for the sample at 0.125, 0.25, and 0.5 mg mL^−1^ of NPs. The measurements were recorded using Spectra Manager 2.09 software (JASCO, Tokyo, Japan) in a 1 cm optical path length cuvette in the range of 300–600 nm, using an excitation wavelength of 265 nm, a 1 nm step resolution, and a 500 nm min^−1^ scan speed.

The hydrodynamic diameter was analyzed after each step of functionalization by Dynamic Light Scattering (DLS). Samples of ZrO_2_-acac NPs, ZrO_2_-acac NPs-APT, and ZrO_2_-acac NPs-HA were prepared at a concentration of 0.05 mg mL^−1^ in distilled water (pH 7.0) and analyzed using a Zetasizer Nano ZS instrument (Malvern Instrument Ltd., Worcestershire, UK) equipped with a He-Ne laser (633 nm) in backscattering mode. The mean hydrodynamic diameter was obtained by averaging three measurements. Additionally, the surface ζ-potential of the samples was investigated with the same instrument using folded capillary cells.

The surface functionalization of ZrO_2_-acac NPs was investigated by Fourier transform infrared spectroscopy (FTIR) using a Thermo-Scientific Nicolet iN10 Infrared Microscope. For the FTIR analysis, the NPs were left to dry on a gold surface used as a background, and an area of 100 × 100 µm^2^ in the range 4000–600 cm^−1^ with a resolution of 16 cm^−1^ was analyzed.

### 2.5. Calculation of ZrO_2_-acac NPs Quantum Yield (QY)

The Quantum Yields (QYs) of the ZrO_2_-acac NPs and ZrO_2_-acac NPs-HA were obtained by the slope of the linear fit curve of the points, with the coordinates absorbance and the integrated PL intensity at the same concentration (0.125, 0.25, and 0.5 mg mL^−1^). Absorption and emission spectra were obtained, respectively, using a Jasco V-730 UV-Vis double-beam spectrophotometer (Jasco Inc., Easton, PA, USA) and a JASCO FP-8200 spectrofluorometer (JASCO, Tokyo, Japan) in the above-described conditions. The QY of the NPs was estimated relative to the L-tryptophan (Trp) used as a standard dye. Instruments were set up in the same conditions, and Trp was analyzed in water at concentrations of 0.0025, 0.05, and 0.010 mg mL^−1^.

### 2.6. Acellular In Vitro ROS Assay

The oxidative potential (OP) of the NPs was preliminarily evaluated by an acellular in vitro ascorbic acid (AA) assay before and after the functionalization [32]. Ascorbic acid solution (100 mM) was used to suspend NPs at the concentration of 0.1 mg mL^−1^ and left with them in stirring conditions for 24 h and 48 h. After the incubation, NPs were separated from the solution by centrifugation at 15,000 rpm, 15 °C, and 10 min; the pellet was discarded, and the supernatant was analyzed by UV-Vis spectroscopy at the fixed wavelength of 266 nm. To exclude possible interference due to the emission of NPs, the supernatant of NPs in the absence of AA was also analyzed.

### 2.7. Cell Culture and Alamar Blue Assay

SAOS-2, a human osteosarcoma cell line with several osteoblastic features, was cultured in a 75 cm^2^ cell culture flask in Dulbecco’s Modified Eagle’s Medium (DMEM) supplemented with 10% Fetal Bovine Serum (FBS), antibiotic solution (streptomycin 100 μg mL^−1^ and penicillin 100 U mL^−1^), and 2 mM L-glutamine. Cells were grown at 37 °C in an incubator containing a humidified atmosphere with 5% CO_2_ and 95% air at passages from 1 to 5.

SAOS-2 cell proliferation was tested by the Alamar Blue assay. After 24 h of seeding, cells at a density of 1 × 10^4^ cells well^−1^ in a 96-well plate (0.32 cm^2^) were treated with ZrO_2_-acac NPs and ZrO_2_-acac NPs-HA at different concentrations (25, 50, 75 μg mL^−1^) for 24, 48, and 72 h. The in vitro cell proliferation was analyzed by using the Alamar Blue assay. Alamar BlueTM reagent (10 *v*/*v*%) was incubated with cells for 4 h at 37 °C in each well, and optical density was measured with a UV-Vis spectrophotometer (Victor X3 Multilabel Plate Reader, PerkinElmer, Waltham, MA, USA) at wavelengths of 570 and 600 nm. The incubation time was selected based on the time cells take to convert resazurin to resorufin. The optical density detected by this assay is proportional to the metabolic activity of live cells. Empty wells (without cells) were used to correct any background interference from the redox indicator.

### 2.8. Internalization Studies by Confocal Microscopy

The internalization of ZrO_2_-acac NPs and ZrO_2_-acac NPs-HA in SAOS-2 cells was performed by confocal laser scanning microscopy (Leica TCS sp8 confocal microscope). To this aim, the cells were cultured on glass slides at a density of 2 × 10^4^ cells slide^−1^ (22 × 22 mm). After 48 h of exposure to ZrO_2_-acac NPs and ZrO_2_-acac NPs-HA (50 μg mL^−1^), the cells were washed three times with PBS and fixed in 4% formaldehyde overnight at 4 °C. Then, the cells were washed three times with PBS and permeabilized with 0.1% Triton X-100 in PBS for 1 h. Then, the cells were washed in PBS, stained with a red phalloidin solution for 1 h, and washed three times. Cells incubated with PBS were used as plate controls. Cell images were acquired with a confocal microscope.

### 2.9. GPX3, P53, and DCFH-DA Assay (ROS Species Detection)

GPX-3 expression was quantified using immunofluorescence analysis. For this purpose, SAOS-2 were plated on glass slides at a density of 2 × 10^4^ cells slide^−1^ (22 × 22 mm). After 24 h of seeding, the cells were incubated with ZrO_2_-acac NPs and ZrO_2_-acac NPs-HA (50 μg mL^−1^) for 48 or 72 h. After cell permeabilization, as previously performed for phalloidin detection, the GPX-3 polyclonal antibody (Bioss) conjugated with Alexa Fluor^®^–488 (1:100 dilution) or the monoclonal rabbit-p53 antibody (Invitrogen) (1:200 dilution) was incubated in each slide at 4° C overnight. After three washes with PBS, the cells were treated with DAPI (10 μg mL^−1^). Finally, the cells were washed three times with PBS and observed by a confocal microscope. Qualitative data were confirmed through image analysis performed using ImageJ software (ImageJ 1.44, which uses Java 1.6 in 64-bit mode) to compare the fluorescence intensity. Fluorescence intensity was calculated by subtracting the cell intensity from the background. To measure ROS species production, SAOS-2 cells, at a density of 1 × 10^4^ viable cells well^−1^ (0.32 cm^2^), were seeded in 96 multi-well plates for 24 h. After seeding, the cells were treated with ZrO_2_-acac NPs and ZrO_2_-acac NPs-HA (50 μg mL^−1^), and the production of intracellular ROS was detected using a fluorescent probe, DCFH-DA. DCFH-DA can penetrate the cell membrane and is hydrolyzed by esterase to form non-fluorescent DCFH, which, instead, is converted into a highly fluorescent DCF by ROS oxidation. After 72 h of exposure to ZrO_2_-acac NPs and ZrO_2_-acac NPs-HA (50 μg mL^−1^), the cells were incubated for 60 min with 10 μM DCFH-DA in HBSS 1% heat-inactivated FBS without calcium, magnesium, and phenol red (56 °C for 30 min). Later on, the cells were stimulated with Fenton’s reagent (H_2_O_2_/Fe^2+^ 2 mM) for 3 h at 37 °C. This oxidant reagent can significantly increase intracellular ROS levels. The DCF signal expressed as fluorescence intensity was measured using a fluorescent microplate reader (BioTeK^®^ Synergy MX, Winnoski, VT, USA) with an excitation wavelength of 485 nm and an emission of 538 nm.

### 2.10. Statistical Analysis

Statistical analyses were undertaken using GraphPad Prism^®^, version 6.00 (GraphPad Software, La Jolla, CA, USA, www.graphpad accessed on 14 June 2022). Data were compared using a Student’s t-test and a one-way ANOVA with a Bonferroni post-test (parametric methods). The results are expressed as the mean ± standard deviation (SD). Values of *p* < 0.05 were considered significant.

## 3. Results and Discussion

### 3.1. Hybrid ZrO_2_-acac NPs Production

In this study, hybrid ZrO_2_-acac NPs were obtained starting from amorphous ZrO_2_−acac sol-gel-derived powders, which have been proposed as a catalyst for environmental purposes thanks to their intrinsic oxidative activity [17].

**Figure 1:**
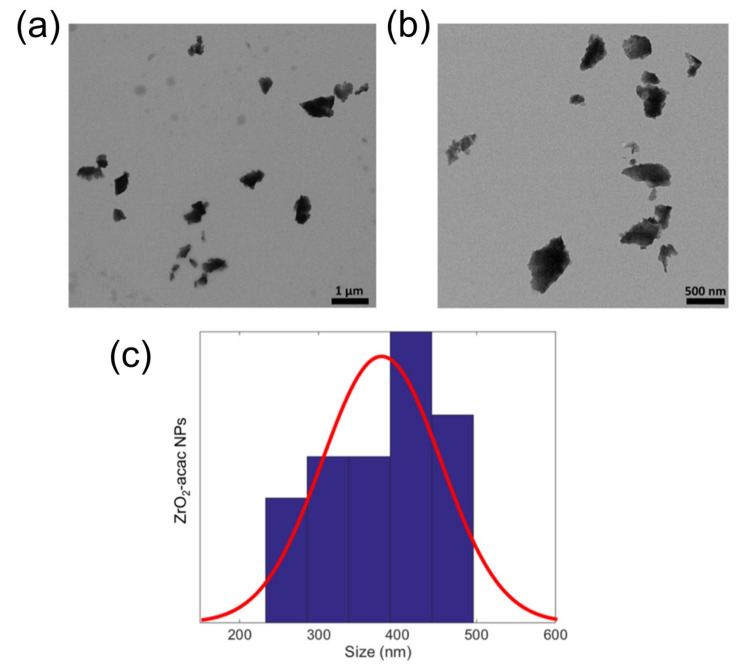
(**a**,**b**) TEM images of the ZrO_2_-acac NPs acquired with two different magnifications. (**c**) TEM size distribution and Gaussian fit.

**Figure 2:**
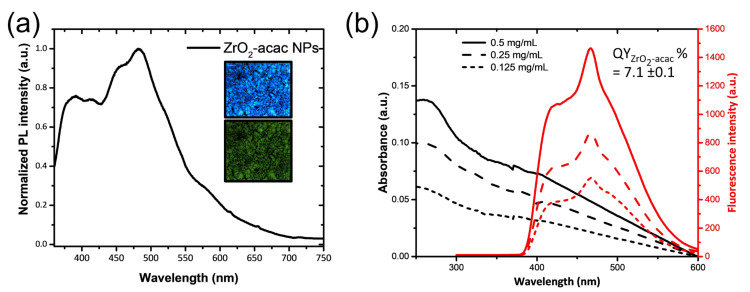
(**a**) Photoluminescence spectrum of ZrO_2_-acac NPs at the excitation wavelength of 325 nm; in the inset, fluorescence microscopy images of ZrO_2_-acac NPs at the excitation wavelengths of 365 nm (on the top) and 470 nm (on the bottom). (**b**) Absorbance and relative photoluminescence of ZrO_2_-acac NPs at different concentrations.

The ability to generate and stabilize the superoxide radical anion on the surface, combined with its optical properties, makes this an interesting material for biomedicine as well. To make hybrid ZrO_2_-acac suitable for biomedical applications, the powder was reduced to the nanoscale (ZrO_2_-acac NPs) using ultrasounds. The ultrasonication process causes microjets in the NPs dispersion, which can wave the particles and the interparticle collision results in the grinding of the matrix. The morphology and size distribution of the ZrO_2_-acac NPs after the treatment were analyzed by TEM (Figure 1). The crushing of the amorphous sol-gel micrometric powders results in NPs with irregular shapes (Figure 1a,b) and an average size of around 420 nm. The optical properties of the ZrO_2_-acac NPs were investigated to demonstrate the potentiality of the material for theranostic applications. The PL spectrum reported in Figure 2a shows a strong and broad luminescence band between 350 nm and 550 nm, respectively, due to the contribution of carbon-related species and oxygen vacancies surrounding the Zr^4+^ ions [21]. Notably, the strong PL in the blue region of the spectrum suggests that the size reduction by ultrasounds did not disrupt the Zr-acac complex, which lowers the formation energy of oxygen vacancies and promotes the formation of O_2_^•−^ radicals [26]. It should be noted that, as opposed to pure ZrO_2_, whose band gap is higher than 5 eV, ZrO_2_-acac has a broad absorption band extended in the visible range (Figure 2b) related to the organic complexes and the defective amorphous structure. In the inset of Figure 2a, the fluorescence of the NPs under two different excitation wavelengths is shown: when the NPs are excited with a wavelength of 365 nm, the emission of the sample is focused in the blue region of the spectrum, while, under an excitation wavelength of 470 nm, the contribution of the green region increases. The quantum yield of ZrO_2_-acac NPs (QY_ZrO_2_-acac NPs_) was estimated by comparing its photoluminescence emission to that of the Trp used as a reference standard of a known quantum yield (15 ± 1%) [33], using Equation (1):

(1) $${\frac{{\mathrm{QY}}_{{\mathrm{ZrO}}_{2}-\mathrm{acac}\;\mathrm{NPs}\;}}{n_{H2O}^{2}\alpha_{{\mathrm{ZrO}}_{2}-\mathrm{acac}\;\mathrm{NPs}}}|}_{\lambda_{265\;\mathrm{nm}}}={\frac{{\mathrm{QY}}_{\mathrm{Trp}}}{n_{H2O}^{2}\alpha_{\mathrm{Trp}}}|}_{\lambda_{265\;\mathrm{nm}}}$$

where *n* is the refractive index of water and α represents the ratio between the integrated PL intensity and the absorbance at the fixed wavelength λ = 265 nm. The coefficient α_ZrO_2_-acac NPs_ was (3.8 ± 0.6) × 10^5^ (Figure S1a), calculated as the slope of the linear regression from the plots of integrated PL intensity versus absorbance for the concentration of NPs of 0.125 mg mL^−1^, 0.25 mg mL^−1^, and 0.5 mg mL^−1^ (Figure 2b). The coefficient of Trp in water α_trp_ was (1.1 ± 0.2) × 10^6^. The quantum yield of the ZrO_2_-acac NPs was estimated to be 7.3 ± 0.1%, showing that the ZrO_2_ NPs can be a promising material for label-free cellular imaging and theranostic applications. 

### 3.2. Functionalization and Characterization of ZrO_2_-acac NPs

**Figure 3:**
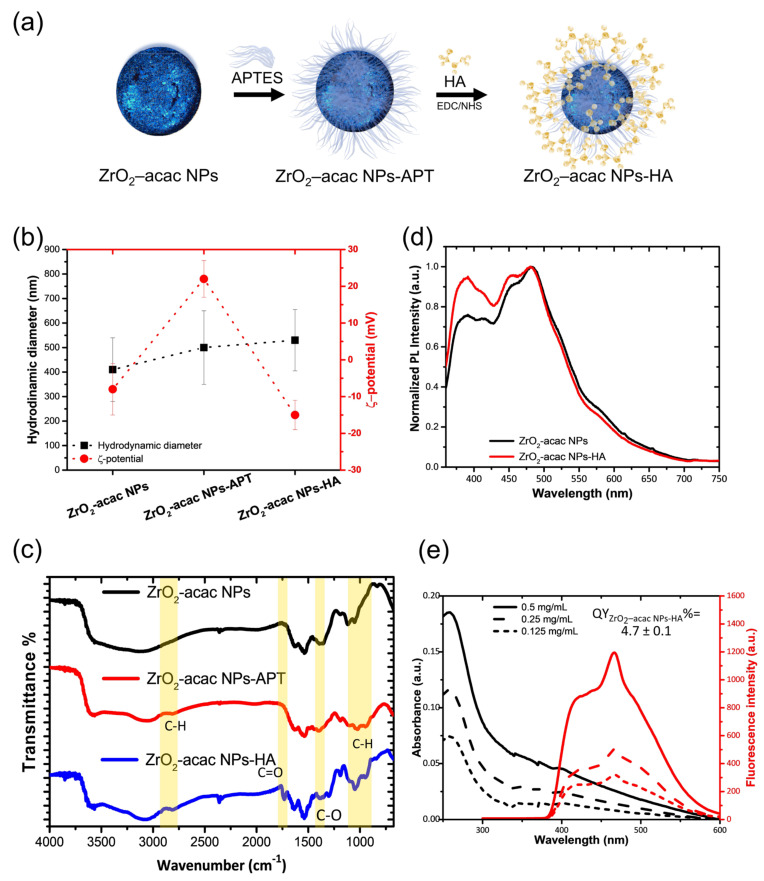
(**a**) Functionalization scheme. (**b**) DLS characterization of ZrO_2_-acac NPs, ZrO_2_-acac NPs-APT, and ZrO_2_-acac NPs-HA. (**c**) FTIR analysis of NPs after each functionalization step. (**d**) Photoluminescence spectra of the NPs before and after the functionalization with hyaluronic acid at the excitation wavelength of 325 nm. (**e**) Absorbance and relative photoluminescence of ZrO_2_-acac NPs-HA at different concentrations. Results are expressed as the mean ± s.d. (*n* ≥ 3).

The surface modification of NPs allows for the introduction of new biological functions for specific antigen targeting. The surface of ZrO_2_-acac NPs was modified with hyaluronic acid (HA) to target osteosarcoma (OS) cells. The hyaluronic acid receptor (CD44) overexpressed on OS cells is capable of binding HA on the ZrO_2_-acac NPs and favors the targeted uptake of NP in the cells [34]. ZrO_2_-acac NPs were functionalized according to the scheme in Figure 3a. The NPs were modified with the amino-silane APTES, which provides the surface with terminal amino groups (−NH_2_), which are further useful for the covalent immobilization of HA by EDC and NHS. The size and the ζ-potential of the system were monitored after each step of functionalization by DLS (Figure 3b). The physicochemical characterization revealed a mean diameter of the NPs of 410 ± 150 nm before the modification, according to the TEM size distribution. The ζ-potential was −8 ± 5 mV due to the presence of acac moieties on the surface. After both APTES and HA functionalization, a slight increment of size was observed (500 ± 150 nm) due to a larger hydration shell surrounding the NPs. The presence of the −NH_2_ groups on the surface of ZrO_2_-acac NPs-APT was confirmed by a positive surface charge (20 ± 6 mV), which turned to −15 ± 4 mV after the modification with HA.

FTIR characterization was performed within the wavelength of 4000−600 cm^−1^ to evidence both the presence of the acac ligand in the ZrO_2_-acac and the bond formation in the functionalized NPs (Figure 3c). The FTIR spectra show the characteristic vibrations of –OH groups at about 3340 cm^−1^ ($\nu_{O-H}$) and 1626 cm^−1^ ($\delta_{HOH}$). The Zr-O-Zr-associated peak lies in the fingerprint region below 600 cm^−1^, as known from the literature, and therefore cannot be appreciated in the graph. The C=C stretching ($\nu_{C=C}$) of enol groups at 1533 cm^−1^ and the peaks at 1376 cm^−1^ and 1283 cm^−1^, corresponding to the stretching of the delocalized C−O bond ($\nu_{C-O}$), confirm that the ultrasound treatment did not disrupt the coordination bond between acac and ZrO_2_ [17,33]. In the FTIR spectra of the ZrO_2_-acac NPs-APT, peaks related to the stretching vibration of C–H ($\nu_{C-H}$) in the alkyl chain of the APTES appeared at 2923 cm^−1^ and 2950 cm^−1^, alongside a peak at 1027 cm^−1^, corresponding to the Si-O bond stretching. ($\nu_{\mathrm{Si}-O}$). The functionalization with HA was confirmed by the presence of the peaks at 1740 cm^−1^ and 1310 cm^−1^, which were related to the stretching mode of the ester ($\nu_{C-O}$) in the HA.

Figure 3d shows that the ZrO_2_-acac NPs-HA preserves the PL emission after the functionalization, even if a weak decrease in the intensity was observed in the blue region spectrum. The quantum yield was estimated as described in Section 3.1, using Trp as a standard dye and equation 1. The absorbance and emission spectra of the ZrO_2_-acac NPs-HA at the concentrations of 0.125, 0.25, and 0.5 mg mL^−1^ follow the spectral shape of the unmodified NPs (Figure 3e). The coefficient α_ZrO_2_-acac NPs-HA_ was (7.1 ± 0.2) × 10^5^, corresponding to a QY of 4.7 ± 0.1%, which is slightly lower than the QY of bare ZrO_2_-acac NPs (Figure S1b).

**Figure 4:**
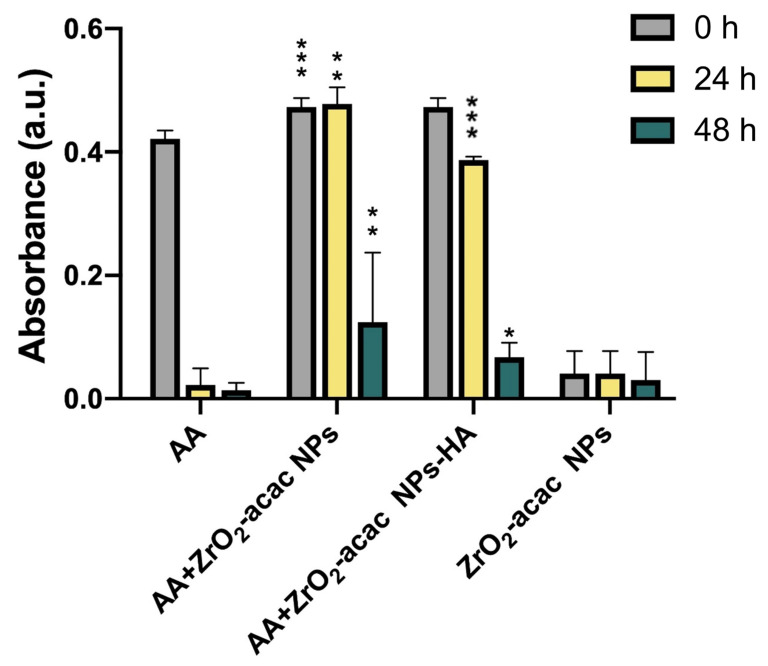
Acellular in vitro AA assay for evaluating the oxidative potential of bare and functionalized NPs at a concentration of 0.1 mg mL^−1^ after 0, 24, and 48 h. Results are expressed as the mean ± s.d. (*n* ≥ 3). The level of significance was set at probabilities of * *p* < 0.05, ** *p* < 0.01, and *** *p* < 0.001. Non-significant data are not reported.

In a previous work [17], the ability of the hybrid ZrO_2_-acac to generate ROS was demonstrated by EPR analysis and attributed to partial electron transfer processes between acac and Zr due to the presence of O_2_ acting as a conduction band electron scavenger hindering the fast charge recombination. Before testing the effects of functionalized NPs in the OS cells, we performed a qualitative ascorbic acid assay to evaluate the residual oxidative potential (Figure 4). The assay was carried out with 0.1 mg mL^−1^ of ZrO_2_-acac NPs or ZrO_2_-acac NPs-HA dispersed in a solution of ascorbic acid (AA). Since the AA absorbance decreases upon oxidation by molecular oxygen, we measured the absorbance of AA at 266 nm to investigate whether the developed NPs played a role in the oxidation process of AA. The AA oxidation was delayed up to 48 h when the molecule was dispersed in the presence of both ZrO_2_-acac NPs and ZrO_2_-acac NPs-HA, thanks to the ability of the NPs to interact and subtract the molecular oxygen to the oxidation process. This result shows that ZrO_2_-acac NPs and ZrO_2_-acac NPs-HA are promising ROS-generating materials for the simultaneous treatment and imaging of OS cells.

### 3.3. Biological Effects of ZrO_2_-Based NPs: Studies of the Cytotoxicity, Uptake, and Induction of Oxidative Stress

Several current studies in the field of cancer therapy are focused on compounds or materials that are able to induce antiproliferative and apoptotic effects by increasing the production of ROS in cancer cells [35]. To date, OS constitutes the most aggressive bone cancer, and it is often refractory to standardized chemotherapy regimens. The effectiveness of chemotherapy, however, has suffered from a range of confounding factors, including systemic toxicity due to lack of specificity, rapid drug metabolism, and multidrug resistance [36] The urgency of targeted and customized treatments for improving a patient’s quality of life has promoted the development of nanomedicine approaches, especially NP-based treatments. While conventional treatments lack selectivity and are associated with painful side effects, the main goal of nanomedicine is to target cancer cells, reducing systemic toxicity and improving patients’ quality of life [37].

**Figure 5:**
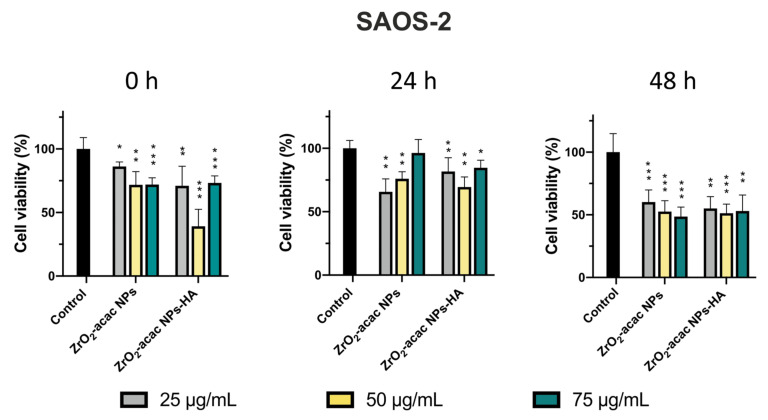
Cytotoxicity test performed with the Alamar Blue assay after 24, 48, and 72 h of incubation of SAOS-2 cells with ZrO_2_-acac NPs and ZrO_2_-acac NPs-HA at 25 (grey), 50 (yellow), and 75 (green) μg mL^−1^. The level of significance was set at probabilities of * *p* < 0.05, ** *p* < 0.01, and *** *p* < 0.001. The results are expressed as the mean ± s.d. (*n* ≥ 3). Non-significant data are not reported.

Here, we tested the efficacy of ZrO_2_-acac NPs and ZrO_2_-acac NPs-HA on SAOS-2 cell proliferation and oxidative stress induction. Preliminary studies concerning the stability of NPs in water and cell culture media were performed, incubating NPs in these two environments for up to 24 h and then analyzing the hydrodynamic diameters of NPs by DLS. The measurements demonstrated the stability of ZrO_2_-based materials in the environments under investigation (Figure S2). We performed cytotoxicity tests using the Alamar Blue assay to investigate the proliferation of SAOS-2 cells starting with 1 × 10^4^ cells/well. After 24, 48, and 72 h of incubation with ZrO_2_-acac NPs and ZrO_2_-acac NPs-HA at different concentrations (25–75 μg mL^−1^), the cell optical density was measured by a UV-Vis spectrophotometer at 560–600 nm. Since the fluorescence intensity of resorufin is proportional to the cell metabolic activity, we measured it to quantify the cell viability and cytotoxicity of the developed NPs. Both ZrO_2_-acac NPs and ZrO_2_-acac NPs-HA, at a concentration of 50 μg mL^−1^, exerted the best effect in terms of proliferation inhibition after 24 and 48 h of cell–NPs interaction. A remarkable cytotoxic effect in terms of decreased SAOS-2 proliferation compared to the control was observed after 72 h of cell exposure to ZrO_2_-acac NPs and ZrO_2_-acac NPs-HA at all the tested concentrations (Figure 5). HA is a well-recognized biocompatible material and is not expected to increase the cytotoxicity of the developed NPs but is likely to improve their internalization. The cytotoxicity induced by ZrO_2_-acac NPs is preferentially ascribed to the release of self-generated ROS inside cancer cells. Due to this, the effects of ZrO_2_-acac NPs-HA and ZrO_2_-acac NPs on the cell viability after 24, 48, and 72 h are comparable. Preliminary studies on healthy fibroblast L929 treated with ZrO_2_-acac NPs at different concentrations (25–100 μg mL^−1^) were also performed to investigate the NP-induced toxicity in non-malignant cells. L929 cells did not show any toxicity upon incubation with ZrO_2_-acac NPs at concentrations of 25 and 50 μg mL^−1^ for 72 h of incubation (Figure S3), thus suggesting a selective effect of these NPs on cancer cell lines. The cytotoxic effect of ZrO_2_-acac NPs on SAOS-2 and the negligible toxicity on L929 healthy cells at 50 μg mL^−1^ suggest that this system acts selectively towards cells with excessive oxidative stress, such as cancer cells. Moreover, to further increase the selectivity of the proposed nanosystem, we modified ZrO_2_-acac NPs with HA targeting the CD-44 overexpressed on SAOS-2 cells. The proposed strategy is designed to enhance the uptake of the NPs and concentrate their effect on the targeted cells, thus increasing oxidative stress and promoting apoptosis in SAOS-2 cells.

**Figure 6:**
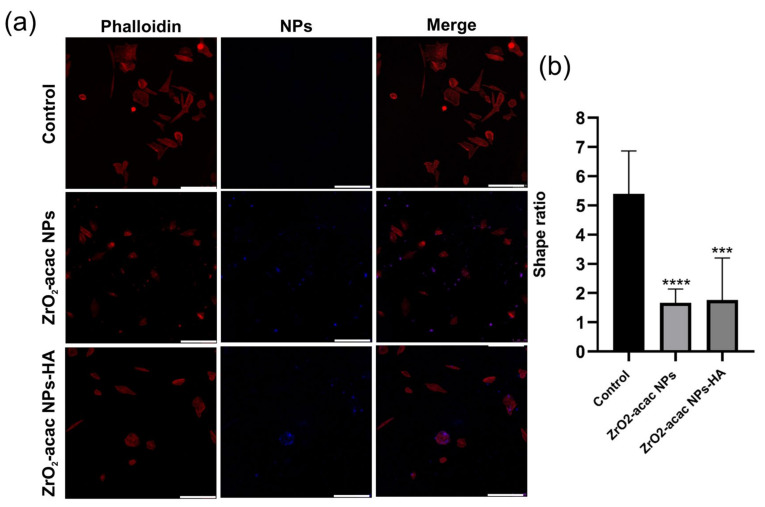
(**a**) Confocal analysis of SAOS-2 cells (red) incubated with ZrO_2_-acac NPs and ZrO_2_-acac NPs-HA (blue) for 48 h. Cell membranes were stained with red phalloidin. Both of the developed formulations interacted with cancer cells, altering their viability, as can be seen by changes in the SAOS-2 morphology and cell density. The scale bar is 250 µm. (**b**) Cell shape index calculated by the image analysis of the SAOS-2 cells incubated with ZrO_2_-acac NPs and ZrO_2_-acac NPs-HA. The images are representative of four experiments. The level of significance was set at probabilities of *** *p* ≤ 0.0002 and **** *p* ≤ 0.0001 vs. control.

Cancer cells were incubated with NPs for 48 h and then investigated by confocal fluorescence microscopy to monitor the intracellular uptake of ZrO_2_-acac NPs and ZrO_2_-acac NPs-HA (Figure 6). SAOS-2 cells were mainly characterized by a spindle-shaped morphology with an oval flat nucleus [38]. This peculiar feature is gradually lost by cancer cells during apoptosis due to a morphological change driven by the cytoskeleton and characterized by cell shrinkage and apoptotic bodies [39]. To study the uptake of our nanosystem, the cells were incubated with 50 μg/mL of both the developed NPs and left to interact for 48 h. Then, the cell membranes were stained with red phalloidin (Figure 6a), whereas NPs were characterized by intrinsic emissive properties (blue) and did not need further modifications. The photoluminescence of ZrO_2_-acac NPs enabled the performing of internalization studies without labeling approaches, which may negatively affect cell–NPs interactions. The obtained results demonstrated the presence of ZrO_2_-acac NPs and ZrO_2_-acac NPs-HA interacting with SAOS-2 cells after 48 h of incubation, as shown by the presence of blue spots in the channel of NPs (Figure 6a). The main difference in the cell incubation of ZrO_2_-acac NPs and ZrO_2_-acac NPs-HA is their distribution on the cell monolayer, which is influenced by the presence of the targeting agent HA. ZrO_2_-acac NPs display a random distribution and interaction with SAOS-2 cells, with only a few spots of NPs attached to the cell membrane (red) (Figure 6a). The absence of a pattern distribution at either the cell membrane or inside the cells may indicate an unspecific interaction of ZrO_2_-acac NPs with SAOS-2 cells. On the contrary, ZrO_2_-acac NPs-HA are mainly found to be attached to the cell membrane and internalized, where the major HA receptor CD44 is expressed, as confirmed by the overlapping signals of NPs (blue) and cells (red). A higher magnification of the images of SAOS-2 cells incubated with ZrO_2_-acac NPs and ZrO_2_-acac NPs-HA is shown in the Supplementary Materials ( Figure S4) to further highlight this point. Based on the different pattern distribution observed in Figure 6a, the modification of ZrO_2_-acac NPs with HA helped the nanosystem orient itself towards the cell membrane, increasing the likelihood of NPs’ penetration. Furthermore, differences in both cell density and morphology are evident before (control) and after the incubation with ZrO_2_-acac NPs and ZrO_2_-acac NPs-HA, thus supporting the cytotoxicity effect of the developed formulation. The spindle-shaped features of SAOS-2 cells (control) are replaced by a rounded morphology upon incubation with ZrO_2_-acac NPs as a consequence of the apoptotic process. For each cell, the shape ratio was obtained by approximating the cell to an ellipse and measuring major and minor semi-axes (ImageJ 1.44 software). Then, the ratio between the major and minor semi-axes was calculated, and mean values of more than 10 cells per sample were reported in Figure 6b (*** *p* ≤ 0.0002 and **** *p* ≤ 0.0001 vs. control). The shape ratio in the morphology of the cells treated with ZrO_2_-acac NPs and ZrO_2_-acac NPs-HA comes closest to 1, which is the index of cells with round bodies (apoptosis) compared to those in the control with a better spreading. The morphological change and reduction in cell density are more evident for the cell culture treated with ZrO_2_-acac NPs-HA, confirming the higher toxicity of ZrO_2_-acac NPs-HA compared to ZrO_2_-acac NPs, as already observed by cytotoxicity studies within 24 h (Figure 5).

To evaluate the impact of ZrO_2_-acac NPs and ZrO_2_-acac NPs-HA on cell oxidative stress, glutathione peroxidase (Gpx)-3 expression and ROS levels were analyzed and quantified. The Gpx-family (Gpx1–3 and 4) is an antioxidant enzyme class that is able to scavenge free radicals by reducing hydrogen peroxide (H_2_O_2_) lipid levels, helping to prevent membrane peroxidation and maintaining the intracellular redox balance [40]. Gpx3 plays a crucial role as a tumor suppressor, decreasing ROS levels and protecting cells from genetic mutations and protein oxidation. Previous results demonstrated that Gpx3 suppression is induced by a significant increment of ROS levels [41], which further induces apoptosis through a Caspase-3-dependant mechanism.

**Figure 7:**
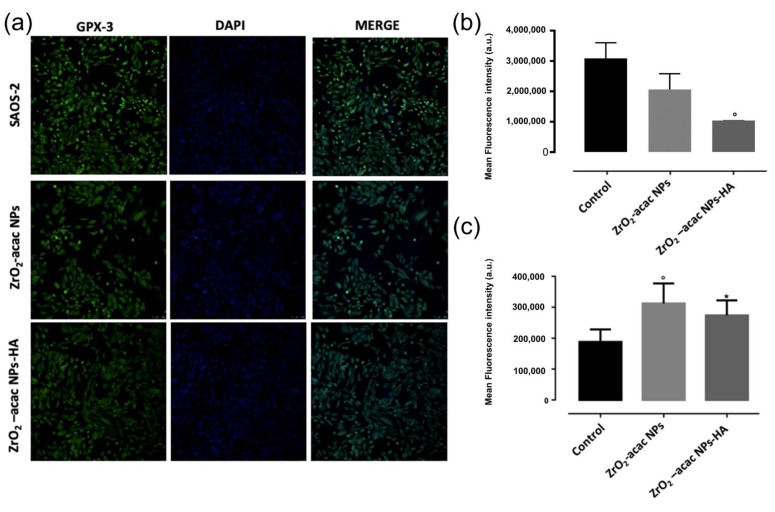
(**a**) Confocal microscopy investigation of Gpx-3 expression on SAOS-2 cells after 48 h of incubation with 50 μg mL^−1^ of ZrO_2_-acac NPs and ZrO_2_-acac NPs-HA, and (**b**) quantification of Gpx expression by ImageJ software. (**c**) ROS generation by DCF-DA assay after incubation of SAOS-2 cells with 50 μg mL^−1^ of ZrO_2_-acac NPs or ZrO_2_-acac NPs-HA. The level of significance was set at probabilities of *p* < 0.05 (*) and *p* < 0.01 (°). The scale bar is 250 µm. The results are expressed as the mean ± s.d. (*n* ≥ 3). Non-significant data are not reported.

Based on this evidence, we suppose that the increased ROS levels induced by the developed NPs may decrease the expression of Gpx-3 enzymes in SAOS-2 cells and promote cell apoptosis. To investigate this mechanism, we incubated SAOS-2 cells with ZrO_2_-acac NPs and ZrO_2_-acac NPs-HA for 48 h at the concentration of 50 μg mL^−1^, which was shown to exert the highest cytotoxicity on the selected cell line. To investigate the expression levels of Gpx3, the cells were further incubated with a Gpx-3-polyclonal antibody conjugated with Alexa-Fluor^®^–488, and cell nuclei were stained with DAPI (Figure 7). Images obtained through confocal microscopy studies and supported by quantitative image analysis have shown a reduced cell fluorescence upon incubation with ZrO_2_-acac NPs and ZrO_2_-acac NPs-HA compared to untreated control cells (Figure 7a,b). These results suggest that both kinds of NPs decreased Gpx-3 expression in SAOS-2 cell through increased ROS levels, which downregulated the expression of antioxidative mechanisms, such as Gpx3. To confirm the activation of cell death mechanisms following higher ROS levels induced by ZrO_2_-acac NPs-HA in SAOS-2, p53 expression was investigated through immunofluorescence analysis. Indeed, as previously reported [42], the suppression of the p53 gene determines an uncontrolled proliferation of cancer cells. By contrast, the expression of p53 associated with increased ROS levels causes a stable cell growth arrest or programmed cell death (apoptosis) by encoding proteins with physiological and biological properties. Here, our results suggested that the cell treatment of Saos-2 cells with ZrO_2_-acac NPs and ZrO_2_-acac NPs-HA for 72 h induced p53 expression (oncosoppressor) compared to the control that does not express the p53 signal (red). The pictures reported in Figure S5 show, in detail, single and merged channels.

The ability of the developed NPs to generate ROS in cancer cells was further assessed using a fluorescent probe DCFH that is converted into the highly fluorescent counterpart DCF upon oxidation by ROS. SAOS-2 cells were incubated with 50 μg mL^−1^ of ZrO_2_-acac NPs and ZrO_2_-acac NPs-HA; then, NPs were removed by washings, and the levels of DCF were measured (Figure 7c). SAOS-2 cells incubated with PBS were considered as a control sample to determine the basal production of ROS in the cells. The generation of ROS in SAOS-2 cells increased upon incubation with both NPs, as demonstrated by the enhanced fluorescence of DCF compared to control cells (Figure 7c). The production of ROS is an intrinsic property of ZrO_2_-acac and is promoted by the presence of oxygen vacancies in the nanomaterial network, which favors and stabilizes the formation of free radicals. This feature is independent of surface modifications, and, therefore, the conjugation of HA to ZrO_2_-acac NPs is not expected to affect the formation of ROS, neither positively nor negatively, as demonstrated by the DCFH-DA assay (Figure 7c). The cancer cells incubated with ROS-generating NPs produced almost twice the amount of ROS shown in the control cells, thus confirming the hypothesis that the Gpx downregulation observed in Figure 7a was caused by increased ROS levels. Overall, the cytotoxicity (Figure 5), Gpx expression (Figure 7a), and ROS quantification (Figure 7b) studies suggest that hybrid ZrO_2_ NPs affect cancer cell viability by increasing ROS levels and shutting the antioxidative machinery down.

## 4. Conclusions

In this work, photoluminescent hybrid zirconia-acetylacetonate nanoparticles (ZrO_2_-acac NPs) that are able to produce reactive oxygen species (ROS) without light activation were proposed for the treatment of osteosarcoma (OS). After the fabrication, ZrO_2_-acac NPs with a mean size of about 420 nm were functionalized with hyaluronic acid (HA) to recognize OS cells binding the overexpressed surface antigen CD44.

Photoluminescence analysis performed after the functionalization procedure demonstrated that the NPs preserved their light emission, characterized by a quantum yield of about 5%.

Biological investigations, including a cytotoxicity test, cellular uptake, and the quantification of protein expression, carried out on OS cells revealed toxic effects already after 48 h of incubation with the developed NPs at a concentration of 50 µg mL^−1^. In particular, the confocal fluorescence microscopy analysis demonstrated an evident change in cell morphology after the treatment. The typical spindle-shaped morphology of OS cells became more rounded as a consequence of the apoptotic process induced by the NPs. Even if NPs were not labeled with a fluorochrome, their natural light emission was visible under UV exposure inside cancer cells. Moreover, the HA targeting improved the interactions of photoluminescent NPs with the cell membrane, which were more consistent with ZrO_2_-acac NPs-HA than bare ZrO_2_-acac NPs, highlighting the ability of HA to target the CD-44 on the OS cells.

The oxidative stress induced by the NPs was demonstrated by investigating the expression of the protein Gpx-3, which is generally affected by an increment of the ROS level. The protein level decreased upon incubation with the ROS-generating NPs, suggesting that ZrO_2_-based NPs increased the oxidative stress in OS cells and promoted their apoptosis.

Based on the results reported in this study, we conclude that the photoluminescent ZrO_2_-acac NPs functionalized with HA can recognize OS cells and induce cell apoptosis by generating and increasing the levels of ROS. Overall, ZrO_2_-acac NPs-HA can be considered an innovative platform for the targeted therapy of cancer that is capable of inducing toxicity without using drugs but generating a high level of ROS.

## Supplementary Materials

The following supporting information can be downloaded at: https://www.mdpi.com/article/10.3390/nano13010054/s1, Figure S1: Plots of the integrated fluorescence intensity at an excitation wavelength of 265 nm versus absorbance at the same wavelength of (a) ZrO_2_-acac NPs and (b) ZrO_2_-acac NPs-HA. The slopes of the fitted lines were used to calculate the QY relative to trp; Figure S2: Stability test of ZrO_2_-acac NPs in H_2_O and cell culture medium (DMEM 10% FBS) after different incubation times (0, 2, 24 h); Figure S3: Cytotoxicity test performed by the Alamar Blue assay after 72 h of the cell culture of L929 cells with ZrO_2_-acac NPs at different concentrations (25–100 µg mL^−1^). Figure S4: Higher magnification of merged images of Saos-2 cells (control and treated with ZrO_2_-acac NPs and ZrO_2_-acac NPs-HA), where it is visible that ZrO_2_-acac NPs-HA are closer to the cell membrane than ZrO_2_-acac NPs. Figure S5: Immunofluorescence analysis of p53 expression in Saos-2 cells treated with 50 µg/mL of ZrO_2_-acac NPs and ZrO_2_-acac NPs-HA compared to the control after 72 h of treatment. The figure shows, in detail, each single channel (cytoskeleton = green, p53 = red, blue = nuclei) and the merged images, as well as the quantification of p53 gene expression as mean fluorescence intensity by ImageJ software.
