# Supplementary material for: ROS-Generating Hyaluronic Acid-Modified Zirconium Dioxide-Acetylacetonate Nanoparticles as a Theranostic Platform for the Treatment of Osteosarcoma
Source: Nanomaterials (Basel). 2022 Dec 22;13(1):54. doi: 10.3390/nano13010054 (PMC9823868; doi:10.3390/nano13010054)
Supplement: Supplementary file 1 [file nanomaterials-13-00054-s001.zip › nanomaterials-2074665-supplementary.pdf]

nanomaterials

Supplementary Materials

ROS-Generating Hyaluronic Acid-Modified Zirconium Dioxide-Acetylacetonate Nanoparticles as a Theranostic Platform for the Treatment of Osteosarcoma

Giovanna Chianese [1,†], Ines Fasolino [2,†], Chiara Tramontano [1,3], Luca De Stefano [1], Claudio Imparato [4], Antonio Aronne [4], Luigi Ambrosio [2], Maria Grazia Raucci [2,*] and Ilaria Rea [1,*]

1 Unit of Naples, National Research Council, Institute of Applied Sciences and Intelligent Systems, 80131 Naples, Italy
2 National Research Council, Institute of Polymers, Composites and Biomaterials, 80131 Naples, Italy
3 Department of Pharmacy, University of Naples Federico II, 80131 Naples, Italy
4 Department of Chemical, Materials and Production Engineering, University of Naples Federico II, 80125 Naples, Italy
* Correspondence: mariagrazia.raucci@cnr.it (M.G.R.); ilaria.rea@na.isasi.cnr.it (I.R.)
† These authors contributed equally to this work.

Citation: Chianese, G.; Fasolino, I.; Tramontano, C.; De Stefano, L.; Imparato, C.; Aronne, A.; Ambrosio, L.; Raucci, M.G.; Rea, I. ROS-Generating Hyaluronic Acid-Modified Zirconium Dioxide-Acetylacetonate Nanoparticles as a Theranostic Platform for the Treatment of Osteosarcoma. *Nanomaterials* **2023**, *13*, 54. https://doi.org/10.3390/nano13010054

Academic Editors: Nicholas Dunne, Helen McCarthy and Tanya Levingstone

Received: 18 November 2022
Revised: 17 December 2022
Accepted: 19 December 2022
Published: 22 December 2022

Copyright: © 2022 by the authors. Licensee MDPI, Basel, Switzerland. This article is an open access article distributed under the terms and conditions of the Creative Commons Attribution (CC BY) license (https://creativecommons.org/licenses/by/4.0/).

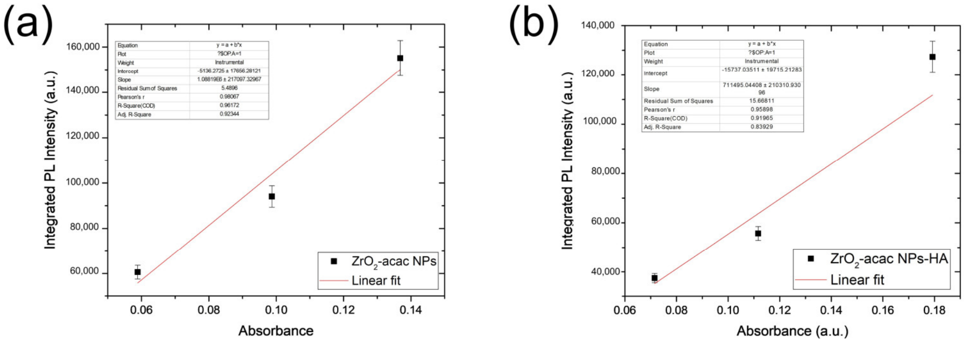

Figure S1. Plots of the integrated fluorescence intensity at excitation wavelength of 265 nm versus absorbance at the same wavelength of (a) ZrO_2-acac NPs and (b) ZrO_2-acac NPs-HA. The slopes of the fitted lines were used to calculate the QY relative to trp.

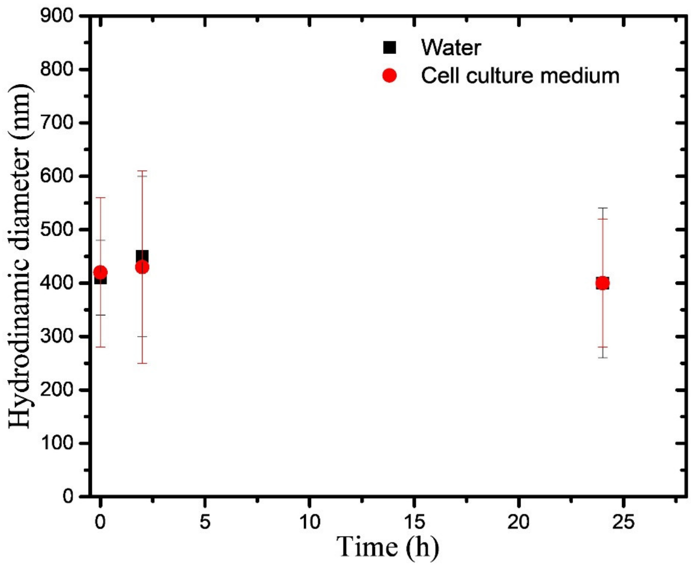

Figure S2. Stability test of ZrO_2-acac NPs in H_2O and cell culture medium (DMEM 10% FBS) after different incubation times (0, 2, 24 h).

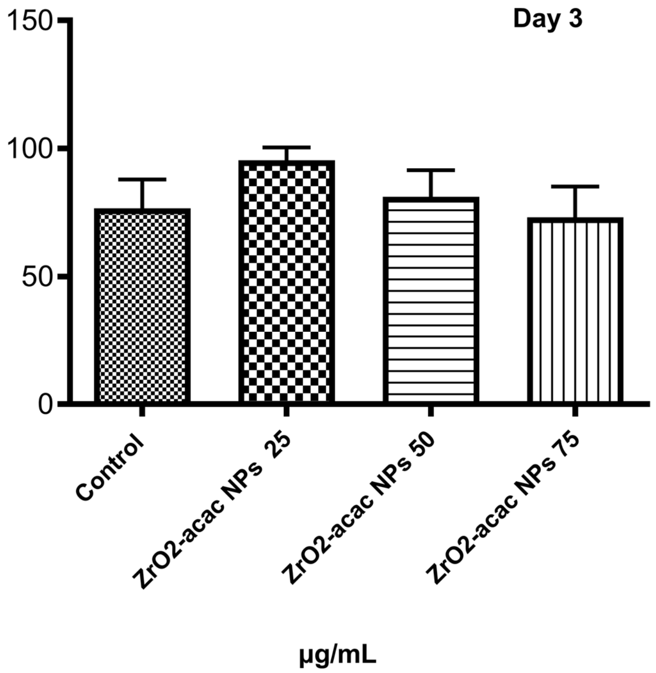

Figure S3. Cytotoxicity test performed by Alamar blue assay after 72 h of cell culture of L929 cells with ZrO_2-acac NPs and ZrO2-acac NPs-HA at different concentrations (25- 75 µg mL^{-1}).

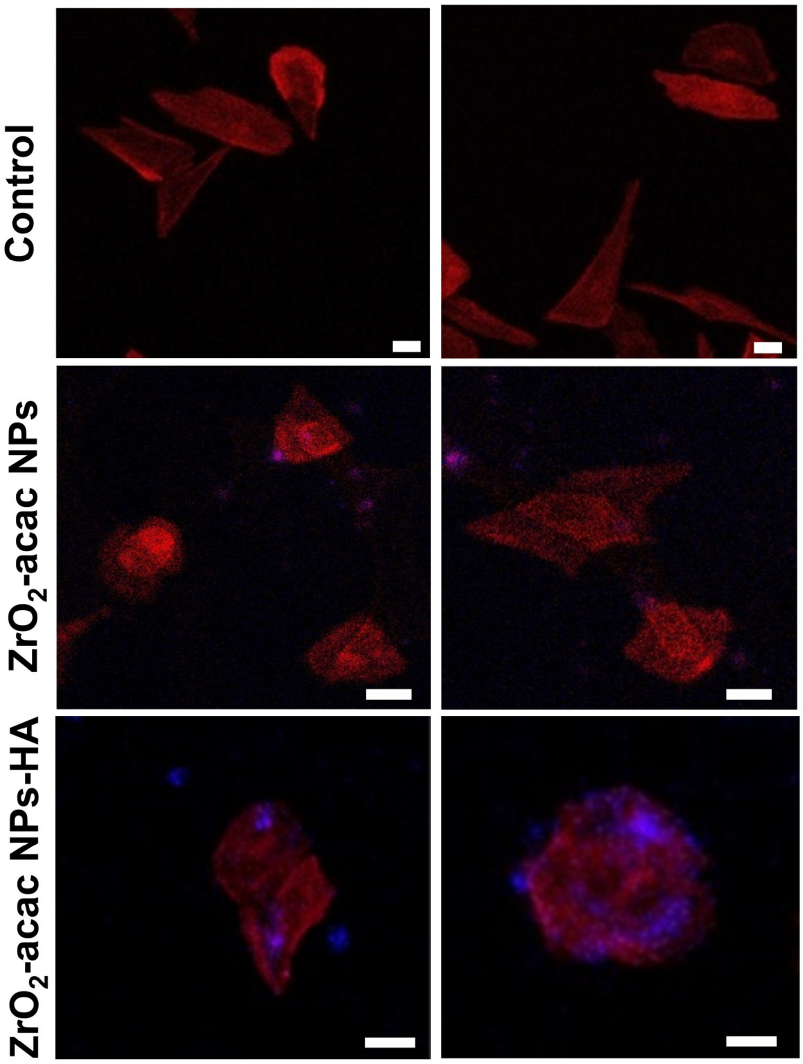

Figure S4. Higher magnification of merged images of Saos-2 cells control and treated with ZrO_2-acac NPs and ZrO_2-acac NPs-HA, where it's visible that ZrO_2-acac NPs-HA are closer to the cell membrane than ZrO_2-acac NPs. The scale bar is 25 μm.

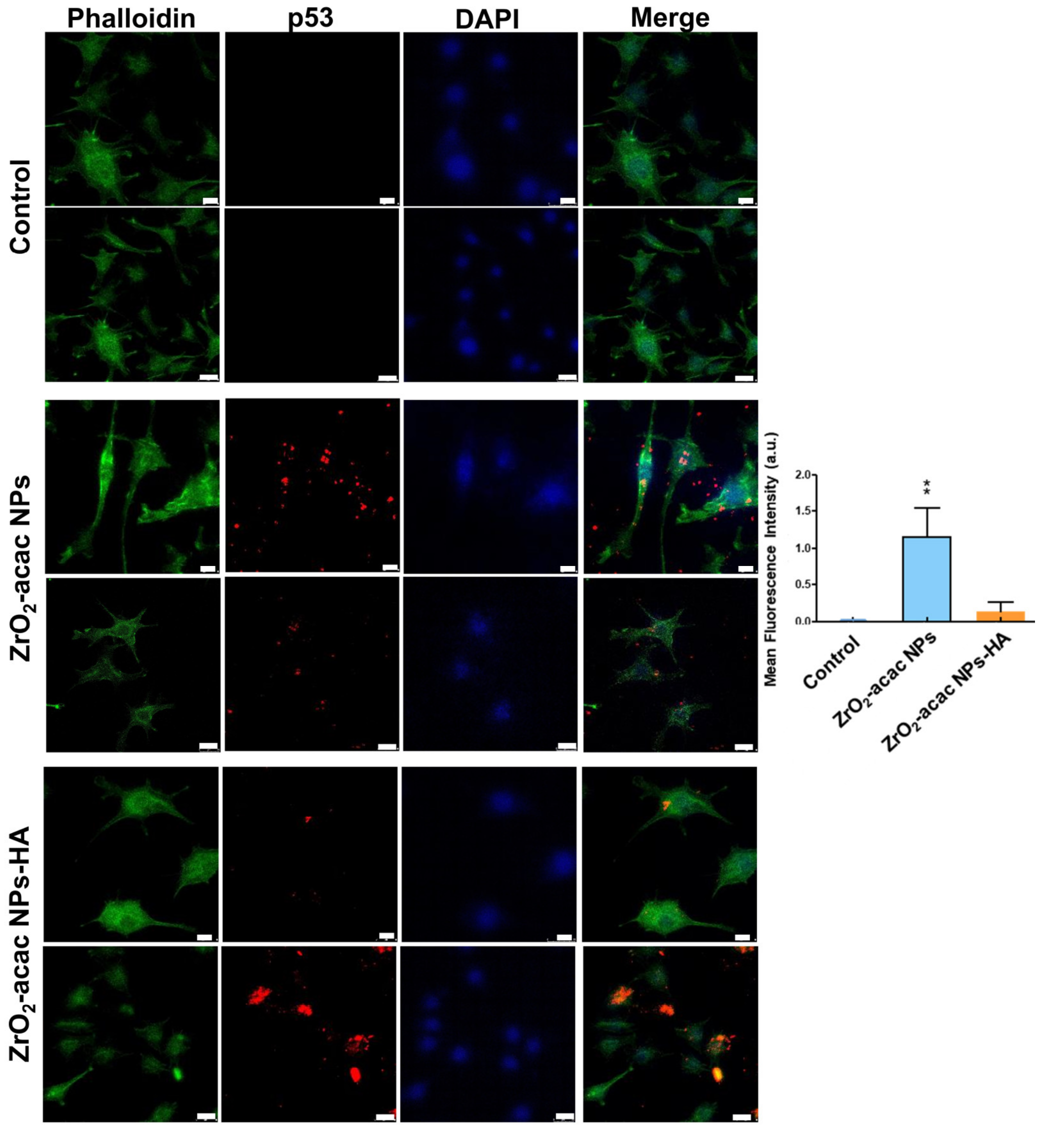

Figure S5. Immunofluorescence analysis of p53 expression in Saos-2 cells treated with 50 µg/ml of ZrO_2-acac NPs and ZrO_2-acac NPs-HA compared to control after 72 h of treatment. Cytoskeleton=green, p53=red, blue=nuclei. The quantification of p53 was done by Image J Software as mean fluorescence intensity study. The scale bar is 25 µm. Results are expressed as mean ± s.d. ($n \geq 3$). The level of significance was set at probabilities of ** $p < 0.01$. Non-significant data are not reported.
